# P-1875. Building Sustainabil-ID: Advancing a New Field of Environmental Stewardship in Infectious Diseases

**DOI:** 10.1093/ofid/ofaf695.2044

**Published:** 2026-01-11

**Authors:** Shreya Doshi, Preeti Jaggi

**Affiliations:** Children's National Health System, Washington DC, DC; Emory University, Atlanta, GA

## Abstract

**Background:**

Healthcare contributes 8.5% of U.S. greenhouse gas emissions (GHGe).Infectious diseases (ID) professionals are uniquely positioned to lead environmental sustainability efforts given collaborative and leadership roles. Sustainabil-ID (Sustainability in ID) is a research collaborative that was launched to formalize this emerging field.

**Figure:**
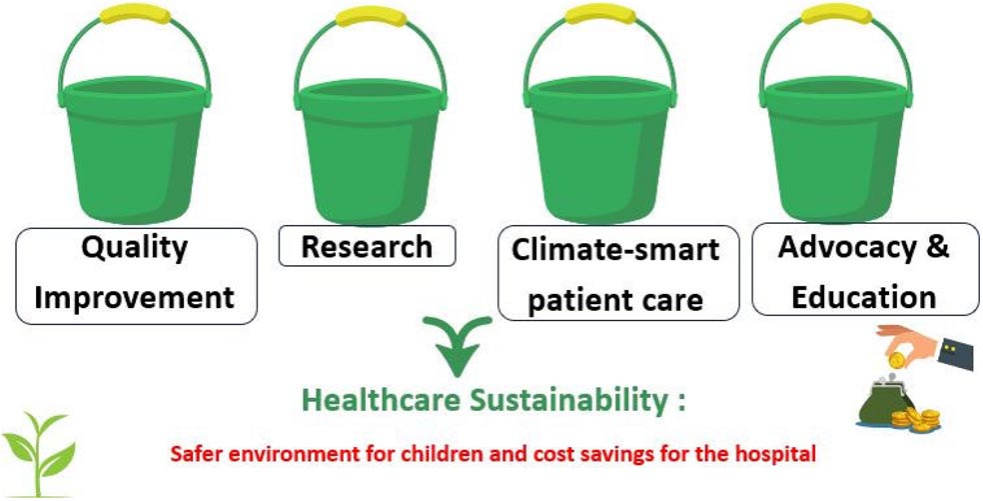
Different Pathways to Sustainability in ID Different Pathways to Sustainability in ID

**Table 1:**
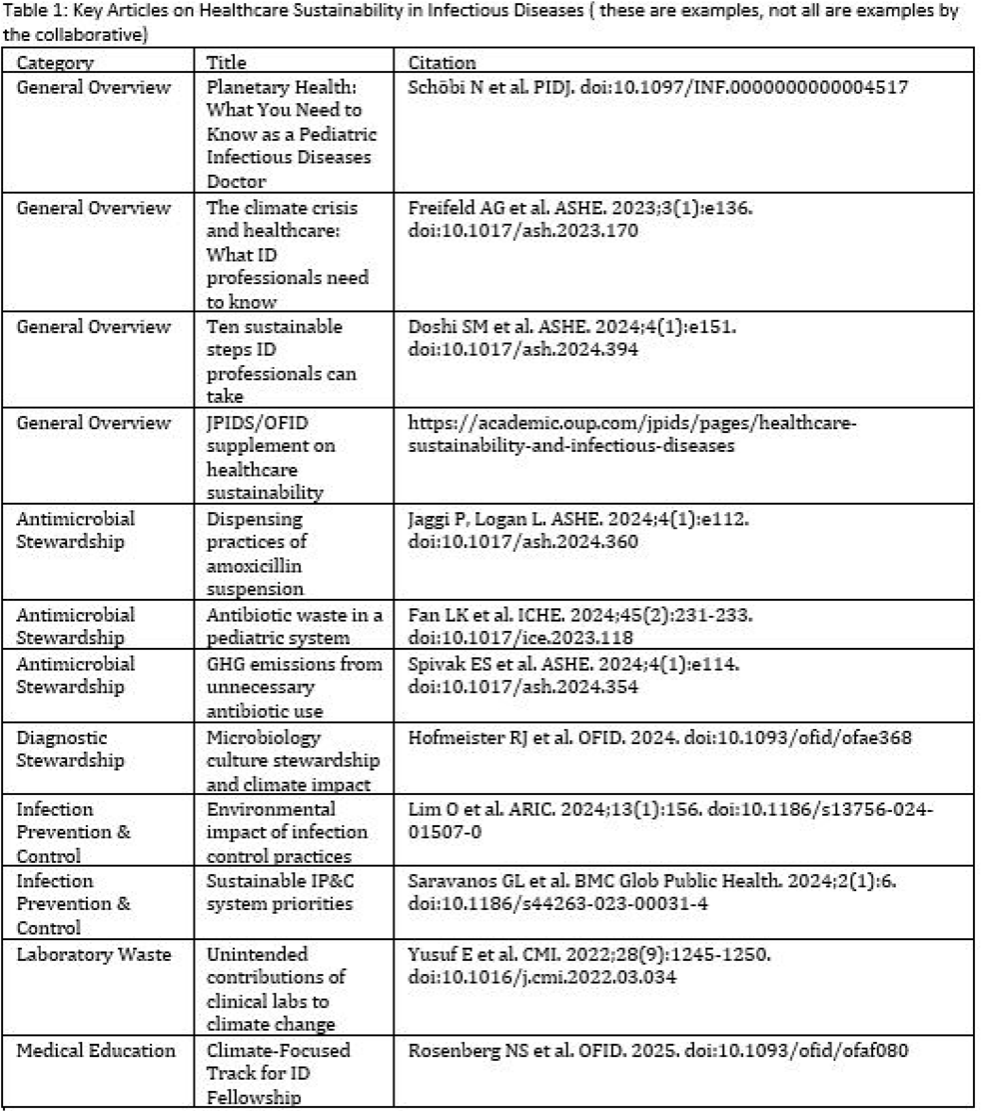
Key Articles on Healthcare Sustainability in Infectious Diseases Table 1: Key Articles on Healthcare Sustainability in Infectious Diseases ( not an all inclusive list)

**Methods:**

We convened a multidisciplinary group of ID clinicians and pharmacists to develop a four-pathway model for integrating sustainability into ID: research, quality improvement, advocacy, and education (Figure 1). Monthly virtual meetings began in 2023. We aligned efforts with national calls to action (e.g., Scope 3 emissions, telemedicine, IV-to-oral switches). A curated repository of tools and literature (Table 1) is discussed in meetings and shared via email with members.

**Table 2:**
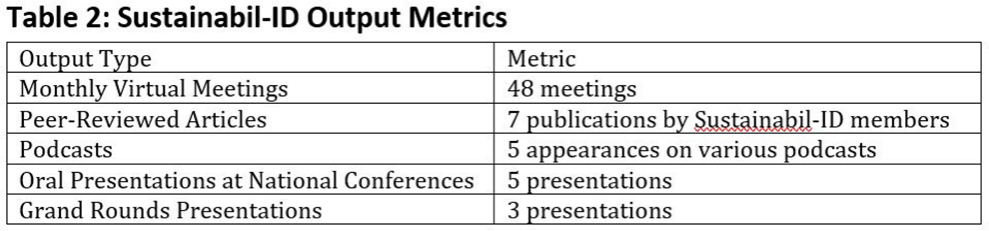
Sustainabil-ID Output Metrics Some metrics that showcase how the field is evolving with more recent publications in the area

**Figure 2:**
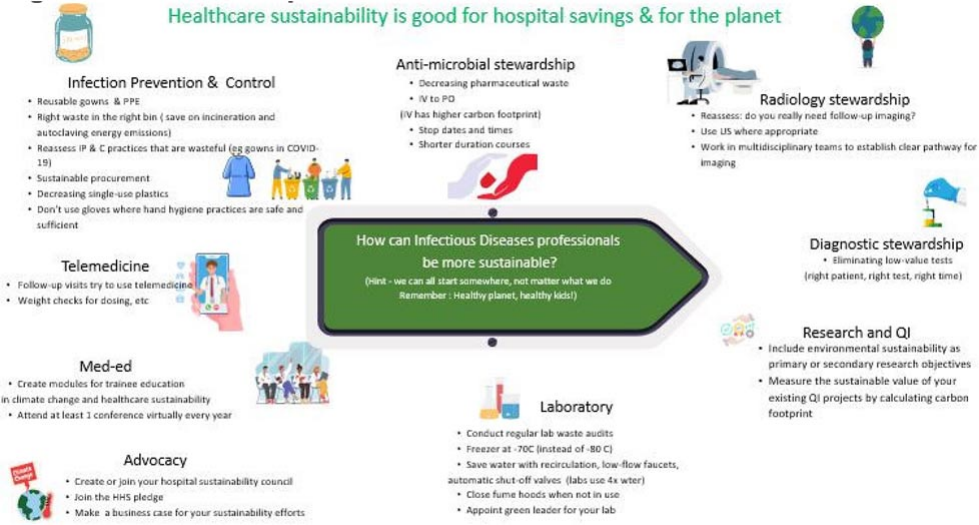
How can professionals be more sustainable? Actionable steps for all providers

**Results:**

The Sustainabil-ID network has grown to 260+ listserv members, with 30–40 attending monthly virtual meetings. Key outputs (Table 2) include peer-reviewed publications on antimicrobial waste, sustainability, and infection prevention. Topics discussed include IV-to-oral conversions, reducing anti-infective waste, single-use plastic reduction, and greening labs (Figure 2). In 2025, Sustainabil-ID became a formal PIDS subcommittee; PIDS also issued a statement on the importance of this topic. The group remains open to pediatric and adult ID professionals globally. Members will present at five national conferences in 2025. Ongoing efforts include curriculum development, multi-site research, and a public-facing resource hub.

**Conclusion:**

Sustainabil-ID defines a new field in infectious diseases focused on reducing healthcare’s greenhouse gas (GHG) emissions. As climate change drives the spread of vectorborne, waterborne, and zoonotic infections, ID professionals must lead sustainability efforts. This interdisciplinary model offers practical strategies for GHG reduction rooted in core ID principles. Broader engagement across pediatric and adult ID is vital to mitigate climate harms and protect public health.

**Disclosures:**

All Authors: No reported disclosures

